# Our Thanks

**Published:** 1875-05

**Authors:** 


*Our Thanks.*

We take this occasion to return our sincere thanks to the Abingdon, Va., Academy of Medicine, for the honor conferred upon us in the appointment as delegate to represent it in the convention of the American Medical Association at Louisville, Kentucky. We regret our inability to attend the Convention, owing to paramount engagements. It would have been an occasion of great pleasure to us, to have met so many old friends, from various parts of the country, and to have met in friendly converse those with whom we hold most agreeable intercourse by correspondence.

We assure our esteemed friends in Abingdon, that we deeply appreciate the honor conferred, in making us their choice as representative in one of the most important assemblages of the day, one whose deliberations were marked by scientific research, of fraternal harmony, and whose work will doubtless bear good fruit, for the benefit of the profession, and the world in general. P.

